# Prolonged and enhanced suppression of thymidylate synthase by weekly 24-h infusion of high-dose 5-fluorouracil

**DOI:** 10.1054/bjoc.2000.1456

**Published:** 2000-11-22

**Authors:** K-H Yeh, S-H Yeh, C-H Hsu, T-M Wang, I-F Ma, A-L Cheng

**Affiliations:** Departments of Oncology, Internal Medicine, National Taiwan University Hospital, 7 Chung-Shan South Road, Tapei, 100, Taiwan; Cancer Research Center, National Taiwan University College of Medicine, Taipei, Taiwan

**Keywords:** thymidylate synthase suppression, high-dose 5-fluorouracil

## Abstract

We have recently demonstrated that HDFL (high-dose 5-FU 2600 mg m–2 week–1 and leucovorin 500 mg m–2 week–1, weekly 24-h infusion) is highly active in the treatment of gastric cancer. To further clarify the possible mechanism underlying the improved activity of HDFL compared with conventional 5-FU regimens, we conducted in vitro studies examining the effect of these regimens on the differential regulation of thymidylate synthase (TS) in NCI-N87, a human gastric cancer cell line. The expected serum concentrations of 5-FU are 100–200 mM (lasting for less than 30 min) and 5–10 mM (lasting for 24 h) for the conventional 5-FU regimens (bolus injection or short intravenous infusion of 5-FU 370–500 mg m–2) and the HDFL regimens, respectively. Western blot analysis revealed that 24-h exposure of NCI-N87 to 2.5–10.0 mM of 5-FU resulted in a dose-dependent depletion of free TS, lasting for more than 24 h. In contrast, 30-min exposure of NCI-N87 to 200 mM of 5-FU resulted in a less than 12-h depletion of free TS. Moreover, 24-h exposure to 5-FU resulted in a higher S-phase blockade and enhanced cytotoxicity. In both modes of 5-FU treatment, the initial rapid depletion of free TS was accompanied by a rapid increment of a higher-molecular-weight TS molecule, suggesting that rapid formation of the ternary complex was the key mechanism of 5-FU action during this period. Northern blot analysis showed that the steady-state mRNA of TS was not affected by either of the schedules. We conclude that 24-h exposure of gastric cancer cells to low concentration of 5-FU resulted in better suppression of free TS, a higher degree of S-phase blockade, and enhanced cytotoxicity compared to 30-min exposure to high concentration of 5-FU. These in vitro results may help explain the improved clinical efficacy of HDFL regimens compared to conventional 5-FU regimens. © 2000 Cancer Research Campaign http://www.bjcancer.com


*British Journal of Cancer* (2000) **83**(11), 1510–1515
© 2000 Cancer Research Campaign
doi: 10.1054/ bjoc.2000.1456, available online at http://www.idealibrary.com on IDEAL®

http://www.bjcancer.com

### Prolonged and enhanced suppression of thymidylate synthase by weekly 24-h infusion of high-dose 5-fluorouracil

**K-H Yeh[1,3], S-H Yeh[3], C-H Hsu[1,3], T-M Wang[3], I-F Ma[3] and A-L Cheng[1–3]**

Departments of [1]Oncology; [2]Internal Medicine, National Taiwan University Hospital, 7 Chung-Shan South Road, Tapei 100, Taiwan; [3]Cancer Research Center, National Taiwan University College of Medicine, Taipei, Taiwan

Summary We have recently demonstrated that HDFL (high-dose 5-FU 2600 mg m–2 week–1 and leucovorin 500 mg m–2 week–1, weekly 24-h infusion) is highly active in the treatment of gastric cancer. To further clarify the possible mechanism underlying the improved activity of HDFL compared with conventional 5-FU regimens, we conducted in vitro studies examining the effect of these regimens on the differential regulation of thymidylate synthase (TS) in NCI-N87, a human gastric cancer cell line. The expected serum concentrations of 5-FU are 100–200 mM (lasting for less than 30 min) and 5–10 mM (lasting for 24 h) for the conventional 5-FU regimens (bolus injection or short intravenous infusion of 5-FU 370–500 mg m–2) and the HDFL regimens, respectively. Western blot analysis revealed that 24-h exposure of NCI-N87 to 2.5–10.0 mM of 5-FU resulted in a dose-dependent depletion of free TS, lasting for more than 24 h. In contrast, 30-min exposure of NCI-N87 to 200 mM of 5-FU resulted in a less than 12-h depletion of free TS. Moreover, 24-h exposure to 5-FU resulted in a higher S-phase blockade and enhanced cytotoxicity. In both modes of 5-FU treatment, the initial rapid depletion of free TS was accompanied by a rapid increment of a higher-molecular-weight TS molecule, suggesting that rapid formation of the ternary complex was the key mechanism of 5-FU action during this period. Northern blot analysis showed that the steady-state mRNA of TS was not affected by either of the schedules. We conclude that 24-h exposure of gastric cancer cells to low concentration of 5-FU resulted in better suppression of free TS, a higher degree of S-phase blockade, and enhanced cytotoxicity compared to 30-min exposure to high concentration of 5-FU. These in vitro results may help explain the improved clinical efficacy of HDFL regimens compared to conventional 5-FU regimens. © 2000 Cancer Research Campaign http://www.bjcancer.com

**Keywords**: thymidylate synthase suppression; high-dose 5-fluorouracil

5-Fluorouracil (5-FU) has been widely used as an anti-cancer drug for more than three decades (Grem, 1996). Its clinical application was further expanded by the introduction of an innovative HDFL regimen in which high-dose 5-FU (2600 mg $m^{-2}$ $week^{-1}$) and leucovorin (500 mg $m^{-2}$ $week^{-1}$) were administered by weekly 24-h infusion (Ardalan et al, 1991). We and others have demonstrated that HDFL, alone or in combination with other anti-cancer drugs, is an effective regimen in the treatment of colorectal cancer (Ardalan et al, 1991; Yeh and Cheng, 1994, 1997; Beerblock et al, 1997; de Gramont et al, 1998) and gastric cancer (Vanhoefer et al, 1994; Hsu et al, 1997; Cheng et al, 1998; Yeh and Cheng, 1998). HDFL appeared to be effective in a substantial portion of patients with advanced colorectal (Weh et al, 1994; Yeh et al, 1997), gastric (Vanhoefer et al, 1994), and breast cancer (Wilke et al, 1996), who had failed to response to or progressed after a conventional-schedule 5-FU regimen. This study sought to further clarify the underlying mechanisms that contribute to the enhanced efficacy of HDFL through in vitro studies of the differential regulation of thymidylate synthase (TS).

Thymidylate synthase (TS) is the target enzyme of 5-FU (Tsujinaka et al, 1992; Peters et al, 1994). Overexpression of TS has been demonstrated to be closely associated with drug-resistance toward 5-FU-based chemotherapy in colorectal cancers (Johnston et al, 1994; 1995) and gastric cancers (Johnston et al, 1995; Lenz et al, 1996; Yeh et al, 1998*a*).

5-FU has a short plasma half-life of approximately 8–14 min (Grem et al, 1991). By 24-h infusion of 5-FU 1000–2000 mg $m^{-2}$, the concentration of 5-FU in the peripheral blood was maintained for 24 h at around 5–10 μM (Fraile et al, 1980; Grem, 1996). However, by conventional bolus injection or short intravenous infusion of 5-FU 370–500 mg $m^{-2}$, the concentrations of 5-FU in the peripheral blood was around 100–200 μM, lasting for less than 1 h (Grem, 1996). Although direct evidence remains lacking, these findings suggest that the extent of inhibition of free TS, one of the main sources of 5-FU drug-resistance, may be different in these two representative schedules of 5-FU. The results of this study provide in vitro evidence that 24-h infusion of high-dose 5-FU may result in a much more durable and effective suppression of TS than conventional 5-FU regimens.

### MATERIALS AND METHODS

### Cell culture of human gastric cancer cells

The human gastric cancer cell line NCl-N87 (ATCC CRL-5822) was obtained from the American Type Culture Collection (ATCC) and maintained in RPMI 1640 cell culture medium (Life

*Received 21 February 2000*
*Revised 12 July 2000*
*Accepted 18 July 2000*

*Correspondence to:* A-L Cheng

Technologies Inc, Rockville, MD, USA) containing 10% fetal bovine serum (Life Technologies Inc).

### Western blot analysis for determination of TS protein expression

Total cellular protein from N87 cells was extracted by lysing the cells at 4°C with lysis buffer containing 0.5% Nonidet P-40, 50 mM Tris (pH 7.4), 150 mM NaCl, 0.1% SDS, 100 mM NaF, 1 mM benzamidine, and 10 μg $ml^{-1}$ each of the following protease inhibitors: trypsin inhibitor, aprotinin, and leupeptin. After centrifugation at 14 000 rpm for 10 min at 4°C, protein concentrations of the supernatants were determined with a Bio-Rad protein assay reagent (Bio-Rad Laboratories, Hercules, CA, USA) by checking absorbance at the wavelength of 595 nm. Forty micrograms of each protein sample was boiled at 95°C for 5 min, then resolved by 12.5% SDS-polyacrylamide gel electrophoresis using the method of Laemmli (1970).

The gels were electroblotted onto a nitrocellulose membrane, incubated with blocking solution containing 5% (w/v) skimmed milk in TBST (20 mM Tris HCl, 150 mM NaCl, 0.05% Tween 20, pH 7.5) for 1 h, and then incubated with the TS106 monoclonal antibody at 1:200 dilution (5 μg $ml^{-1}$) (Chemicon International Inc, Temecula, CA, USA) at 4°C overnight.

After overnight incubation, the membrane was washed thoroughly with TBST. A horseradish peroxidase-conjugated goat anti-mouse immunoglobin was then added as the secondary antibody at 1:5000 dilution and incubated for 1 h at room temperature. The membrane was washed again with TBST. The protein bands were visualized by enhanced chemiluminescence assay reagents (Amersham Pharmacia Biotech Inc, Piscataway, NJ, USA).

### Northern blot analysis for determination of steady-state mRNA of TS

Total cellular RNA was prepared by acid guanidinium thiocyanate-phenol-chloroform extraction using the method of Chomczynski and Sacchi (1987). Briefly, the RNA extraction was conducted with N87 cells treated with each protocol. Cells were lysed directly by adding with 4 ml of the Ultraspec RNA extraction reagent (Biotecx Laboratories Inc, Houston, TX, USA) into each dish. Cell lysate was transferred into polypropylene tubes, and 0.8 ml of chloroform: isoamylalcohol mixture (24:1) was added to accelerate the separation of the two layers after acid phenol extraction (pH 4.0). The upper layer, which contains RNA, was precipitated by isopropanol, and the RNA pellet was dissolved in diethylpyrocarbonate-treated water.

RNA was separated by electrophoresis on 1.2% agarose/formaldehyde gels in the presence of ethidium bromide, and transferred to Hybond-N nylon membrane (Amersham Pharmacia Biotech Inc). Blots were pre-hybridized for 2 h at 42°C in 2% (w/v) blocking agent (Boehringer Mannheim Biochemicals, Indianapolis, IN, USA), 50% deionized formamide, 5X SSC (1X SSC containing 150 mM NaCl, 15 mM sodium citrate, pH7.0), 0.2% (w/v) SDS, 0.1% (w/v) N-lauroyl sarcosine, and 20 μg $ml^{-1}$ poly rC (Sigma Chemical Co, St. Louis, MO, USA). Hybridization was conducted overnight under the same conditions at a probe concentration of $2 \times 10^6$ dpm $ml^{-1}$. Two probes were used in this study. One is human thymidylate synthase (TS) cDNA fragment (496 bp) and the other is human glyceraldehyde-3-phosphate dehydrogenase (GAPDH) cDNA fragment (790 bp). Both cDNA fragments were prepared as described previously (Yeh et al, 1998*b*). The DNA probes were labelled with α-[$^{32}$P]-dCTP using a Rediprime labelling kit according to the manufacturers' protocols (Amersham Life Sciences, Arlington Heights, IL, USA). After hybridization, blots were washed twice in 2× SSC-0.2% (w/v) SDS at room temperature, and twice in 0.1× SSC-0.2% (w/v) SDS at 68°C prior to autoradiography at –80°C.

### Determination of TS expression with '30-min short exposure' vs '24-h prolonged exposure' of 5-FU

### *Effects of 5-FU dose on TS expression*

N87 cells were cultured by splitting and plating $5 \times 10^6$ cells per 10-cm culture dish for collection at 24 h after the start of 5-FU treatment. After the cells were plated and cultured overnight, they were treated by the two representative schedules which were designed to mimic in vivo conditions which occur with conventional bolus injection/short infusion regimens of 5-FU (30-min short exposure) and HDFL regimens (24-h prolonged exposure). The effects of 5-FU dose were tested with both schedules using a series of concentrations of 5-FU. For 30-min short exposure, the 5-FU doses used for treatment were 1 μM, 5 μM, 10 μM, 50 μM, 100 μM, and 200 μM. For 24-h prolonged exposure, the 5-FU doses used for treatment were 0.1 μM, 0.25 μM, 0.5 μM, 1 μM, 2.5 μM, 5 μM, and 10 μM. After 5-FU exposure for the indicated time duration, the medium was removed and cells were washed twice with 1× PBS, and fresh RPMI medium was then replaced. The cells were then harvested at 24 h after the start of 5-FU treatment, respectively.

### *Time-course effects of 5-FU on TS expression*

N87 cells were cultured by splitting and plating $5 \times 10^6$ cells, $2.5 \times 10^6$ cells, and $1.25 \times 10^6$ cells per 10-cm culture dish for collection at or before 24h, at 48 h, and at 72 h after the start of 5-FU treatment, respectively. The cells were treated with either 30-min short exposure of 5-FU at the concentration of 200 μM, or 24-h prolonged exposure at the concentration of 5.0 μM. After 5-FU exposure for the indicated time duration, the medium was removed, and cells were washed twice with 1× PBS, and fresh RPMI medium was then replaced. The cells were harvested at 1, 3, 6, 9, 12, 24, 48, and 72 h after the start of 5-FU treatment, respectively.

### MTT cytotoxicity assay of two modes of 5-FU treatment

The cytotoxicity effects of 5-FU with the two modes of administration were evaluated by an MTT (3-(4,5-dimethylthiazol-2-yl)-2,5-diphenyl) tetrazolium bromide) assay. The growth curve of the N87 cells was determined first in a 96-well culture plate with various numbers of cells. Based on the growth curve, optimal number of cells that would be expected to grow exponentially at 72, 96, and 120 h were plated into each well. The cells were allowed to grow and stabilize overnight before 5-FU was added.

Subsequently, the cells were treated with 24-h prolonged exposure to low-dose 5-FU or 30-min short exposure to high-dose 5-FU with the series of concentrations of 5-FU described above. Each treatment was performed in triplicate. After treatment, cells were washed twice with 1× PBS, then 100 μl fresh medium was added. Cells were allowed to grow for a total of 72, 96, and 120 h after the start of 5-FU treatment, respectively. At the end of the indicated incubation period, the number of viable cells was

© 2000 Cancer Research Campaign 

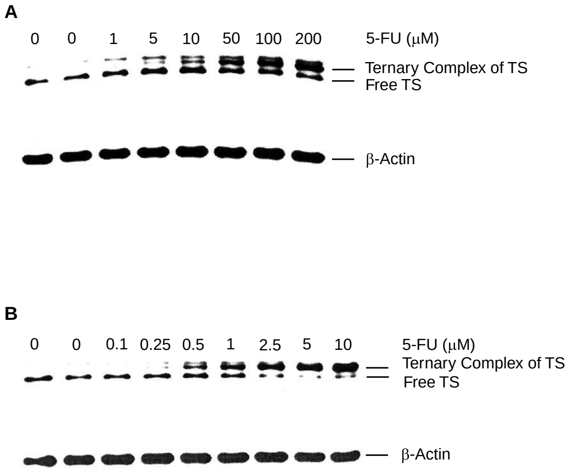

**Figure 1** Effects of 5-FU dose on TS protein expression in the two representative modes of 5-FU treatment. (**A**) After 30-min short exposure of N87 cells to 5-FU (0, 1, 5, 10, 50, 100, and 200 μM), Western blot analysis to determine TS protein expression was performed at 24 h after the start of 5-FU treatment. β-Actin was used as internal control for protein expression. (**B**) After 24-h prolonged exposure of N87 cells to 5-FU (0, 0.1, 0.25, 0.5, 1, 2.5, 5, and 10 μM), Western blot analysis of TS protein expression was performed at 24 h after the start of 5-FU treatment. The lower band denotes the free form of active TS, and the upper band denotes the ternary complex of inactive TS. β-Actin was used as internal control for protein expression

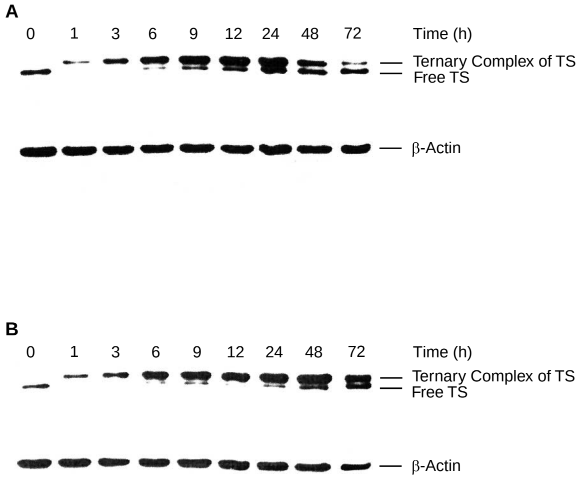

**Figure 2** Time-course effects of 5-FU on TS protein expression. (**A**) After 30-min short exposure of N87 cells to 200 μM of 5-FU, Western blot analysis of TS protein expression was performed at 1, 3, 6, 9, 12, 24, 48, and 72 h after the start of 5-FU treatment. β-Actin was used as internal control for protein expression. (**B**) After 24-h prolonged exposure of N87 cells to 5 μM of 5-FU, Western blot analysis of TS protein expression was performed at 1, 3, 6, 9, 12, 24, 48, and 72 h after the start of 5-FU treatment. β-Actin was used as internal control for protein expression determined by MTT assay. Fifty microliters of MTT reagent (2 mg of MTT $ml^{-1}$ medium) was added to each well followed by incubation at 37°C for 2.5 h. The plate was centrifuged at 2000 rpm for 5 min at 4°C. Medium was removed and 150 μl of DMSO was added to each well. The absorbance of each well was read by a spectrophotometric plate-reader at the wavelength of 492 nm.

### Cell cycle analysis by flowcytometry for the two modes of 5-FU treatment

After completion of 5-FU treatment as described above, N87 cells were trypsinized into single cells, washed once in RPMI, washed once in 1X cold PBS, then fixed in 75% cold ethanol. Cells were stained with 1 ml of propidium iodide solution (2.5 mg of propidium iodide in 50 ml of 1× PBS), and the corresponding cell-cycle status was analysed by FACScan (Becton Dickinson, Mountain View, CA, USA) using the histograms of the program Cell Quest (Becton Dickinson). A total of 10 000 cells were counted by flowcytometry for each cell sample.

### Statistics

The statistical significance of comparison of cell-cycle regulation by the two modes of 5-FU treatment was analysed by the Student's t-test. A $P$ value less than 0.05 was considered significant.

### RESULTS

### Effects of 5-FU dose on TS expression in the two modes of 5-FU treatment

After 30-min short exposure to various concentrations of 5-FU, N87 cells were harvested at 24 h after the start of 5-FU treatment, and TS protein expression was determined by Western blot analysis. As shown in Figure 1A, the expression of ternary complex of TS (upper band) was increased in a dose-dependent manner. However, free TS expression (lower band) remained the same even at the highest 5-FU concentration of 200 μM, which is the estimated serum concentration achieved by bolus injection of a conventional dose of 5-FU.

As shown in Figure 1B, after 24-h prolonged exposure to various lower concentrations of 5-FU, the amount of ternary complex of TS (upper band) was also increased in a dose-dependent manner. In contrast to the high-concentration/short-exposure mode, free TS (lower band) expression remained significantly down-regulated at 24 h by 2.5–10 μM concentrations of 5-FU, which are the estimated serum concentrations achieved by 24-h infusion of high-dose 5-FU.

For further analysis of the time-course effects, 30-min short exposure to 200 μM of 5-FU and 24-h prolonged exposure to 5 μM of 5-FU were selected as the representative conditions for the following experiments.

### Time-course effects of 5-FU on TS expression

As shown in Figure 2A, after 30-min short exposure to 200 μM of 5-FU, the ternary complex of TS (upper band) gradually accumulated up to 24 h, and decreased thereafter. The free TS expression (lower band) was depleted immediately after exposure to 5-FU, but started to recover at 3 h. Complete recovery of free TS was noted at 12 h after the start of 5-FU treatment. In contrast, as shown in Figure 2B, after 24-h prolonged exposure to 5 μM of 5-FU, the ternary complex of TS (upper band) continued to accumulate up to 72 h, and the free TS (lower band) was still depleted at 24–48 h.

### Effects of 5-FU dose on steady-state mRNA of TS

Northern blot analysis showed that steady-state mRNA of TS was not significantly affected by either of the 5-FU treatment schedules, as shown in Figure 3.

*© 2000 Cancer Research Campaign*

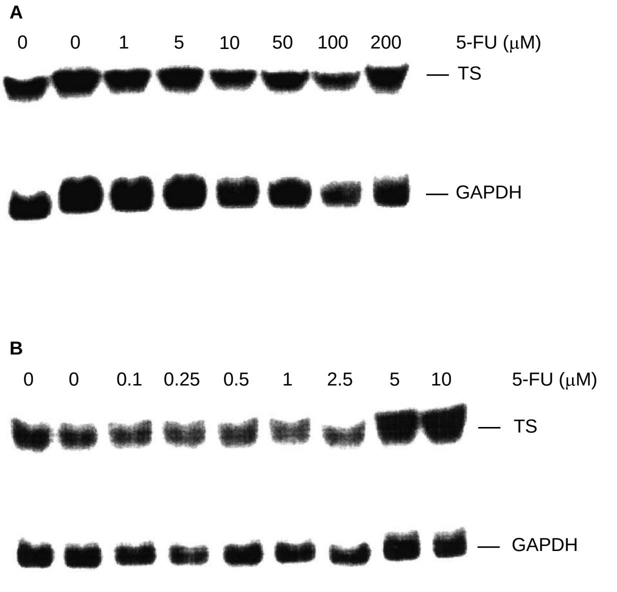

**Figure 3** Effects of 5-FU dose on steady-state mRNA of TS. (**A**) After 30-min short exposure of N87 cells to 5-FU (0, 1, 5, 10, 50, 100, and 200 μM), Northern blot analysis for steady-state mRNA of TS was performed at 24 h after the start of 5-FU treatment. GAPDH was used as internal control for mRNA expression. (**B**) After 24-h prolonged exposure of N87 cells to 5-FU (0, 0.1, 0.25, 0.5, 1, 2.5, 5, and 10 μM), Northern blot analysis for steady-state mRNA of TS was performed at 24 h after the start of 5-FU treatment. GAPDH was used as internal control for mRNA expression

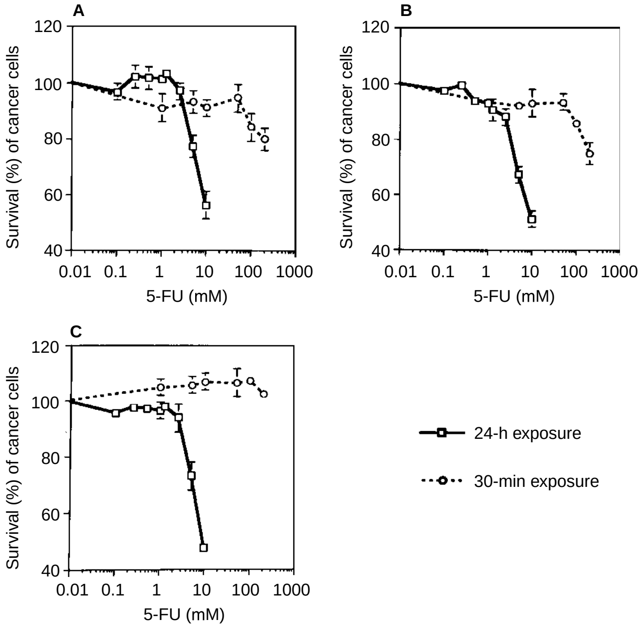

**Figure 4** Comparison of cytotoxicity of the two modes of 5-FU treatment. Y-axis denotes survival fractions (%, mean ± standard deviation) of NCI-N87 gastric cancer cells. X-axis denotes a series of 5-FU concentrations (μM). Results using the two representative modes of 5-FU treatment are shown. (**A**) MTT assay at 72 h after the start of 5-FU treatment. (**B**) MTT assay at 96 h after the start of 5-FU treatment. (**C**) MTT assay at 120 h after the start of 5-FU treatment

### Comparison of cytotoxicity for the two modes of 5-FU treatment

The dose-response curves after 72, 96, and 120 h of cell culture are shown in Figures 4A, 4B and 4C, respectively. The 24-h-exposure mode consistently suppressed cell growth up to 120 h, with an $IC_{50}$ of around 5–10 μM. In contrast, the 30-min-exposure mode had a shorter duration of growth suppression, as evidenced by the regrowth of cells at 120 h. Further, the $IC_{50}$ was more than 200 μM at 72 and 96 h with the 30-min-exposure mode.

### Comparison of cell-cycle regulation by the two modes of 5-FU treatment

As shown in Figure 5, 24-h prolonged exposure to 2.5–5 μM of 5-FU resulted in significantly greater S-phase blockade than 30-min short exposure to 200–400 μM of 5-FU (24-h exposure to 5 μM vs 30-min exposure to 200 μM of 5-FU, $P = 0.005$, t-test). The G0/G1 and G2/M fractions were also more suppressed by the low-dose/prolonged exposure mode.

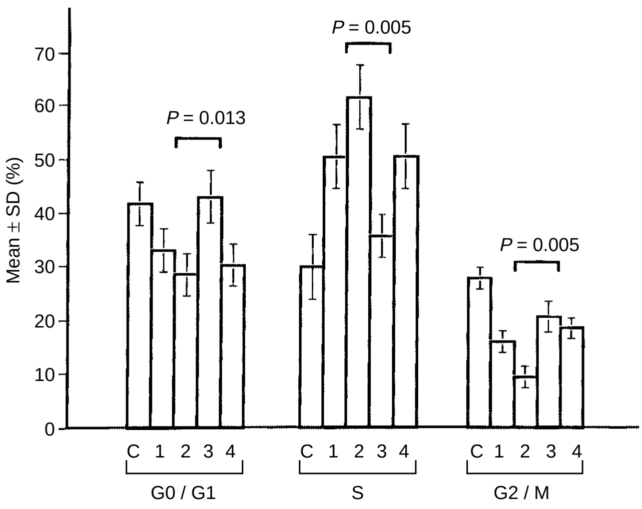

**Figure 5** Comparison of cell-cycle regulation by the two modes of 5-FU treatment. Y-axis denotes the G0/G1, S, and G2/M phases of cell cycle, respectively (%, mean ± standard deviation). X-axis: C = control, no 5-FU treatment; 1 = 24-h treatment with 2.5 μM of 5-FU; 2 = 24-h treatment with 5.0 μM of 5-FU; 3 = 30-min treatment with 200 μM of 5-FU; and 4 = 30-min treatment with 400 μM of 5-FU. Cells were collected for flowcytometric analysis at 24 h after the start of 5-FU treatment

### DISCUSSION

Recently, evidence has accumulated that a weekly 24-h infusion of high-dose 5-FU may improve the response rate and survival time compared with 5-FU bolus regimens (Meta-analysis Group in Cancer, 1998). In a randomized multicentre trial for metastatic colorectal cancer, Kohne et al (1998) reported an overall response rate of 44% and a median survival time of 16 months using a weekly-times-six schedule of infusional 5-FU (2600 mg $m^{-2}$ 24-h infusion) (Kohne et al, 1998). In another randomized study for advanced colorectal cancer, de Gramont et al (1997) reported a significantly better outcome in patients treated by a similar schedule which combined 'bolus plus infusional' 5-FU compared to 'bolus' 5-FU (de Gramont et al, 1997). These results suggest that 24- or 48-h infusion of high-dose 5-FU is more effective than the conventional bolus schedules. However, the pharmacodynamic mechanism responsible for this improved effect is not clear. In this in vitro study, we have demonstrated that a 24-h-infusional 5-FU schedule provides significantly better suppression of TS than the conventional bolus 5-FU schedule.

© 2000 Cancer Research Campaign

The monoclonal antibody, TS106, detects two bands of thymidylate synthase (Johnston et al, 1991). The upper band was not apparent in cells that had not been exposed to 5-FU. However, only the upper band was detected in cells incubated with excess 5-fluorodeoxyuridine monophosphate (FdUMP) or 5, 10-methylene-tetrahydrofolate ($CH_2H_4$PteGlu), suggesting that the upper band represents the ternary complex of TS while the lower band represents the free form of TS protein (Johnston et al, 1991). The free form of TS is an active enzyme and its expression is inversely correlated with the drug sensitivity of several human cancers (Chu et al, 1991*a*; Li et al, 1995). The ternary complex is an inactive form of TS and its activity has been shown to be inhibited by complexing with FdUMP (Johnston et al, 1991; 1992; Grem, 1996). The results of this study indicate that 24-h prolonged exposure to 5–10 µM of 5-FU resulted in more sustained suppression of free TS than 30-min short exposure to 100–200 µM of 5-FU. With the 24-h exposure, suppression of free TS was still evident more than 24 h after the start of 5-FU treatment; however, with the 30-min exposure, suppression of free TS lasted for less than 12 h. The mechanism responsible for the prolonged suppression of TS by the 24-h-infusional schedule remains unclear. TS expression has been reported to be regulated at both a transcriptional (Dolnick et al, 1996; Banerjee et al, 1998) and a translational level (Chu et al, 1991*b*; Chu and Allegra, 1996). In this study, the steady-state expression of the mRNA of TS was not affected by either of the 5-FU treatment schedules. Therefore, translational regulation of TS may have played an important role in prolonged suppression of TS with the 24-h-infusional schedule. In a model of translational regulation of the expression of TS, excess free TS would bind to the regulatory region of TS mRNA, and causes feedback inhibition of TS translation (Chu et al, 1991*b*; Chu and Allegra, 1996). The formation of the ternary complex releases free TS from the regulatory region of mRNA, and therefore results in a rebound of TS translation (Chu et al, 1991*b*; Chu and Allegra, 1996). This model appears to helpfully explain the observations of this study. In the short-exposure mode, free TS was completely depleted during the first hour of exposure to high-dose 5-FU. The absence of free TS then caused increased TS translation, which became apparent at 3 h, and even overshot to a level higher than the control at 24 h after the start of 5-FU treatment. In the prolonged-exposure mode, the new TS synthesized as a result of increased TS translation was very likely depleted by the continual formation of the ternary complex. Higher TS activity, representing an increased amount of free TS, was noted during the S-phase under physiological conditions (Navelgund et al, 1980). Since 24-h prolonged infusion with 5–10 µM of 5-FU resulted in enhanced suppression of free TS, it thus caused more S-phase blockade and enhanced cytotoxicity, as was found in this study.

The optimal dose and schedule of infusional 5-FU remains unclear. The data of the present study suggest that a concentration of 2.5 µM, a level less than half of that achieved by 24-h infusion of 2600 mg $m^{-2}$ of 5-FU still effectively suppressed free TS (Figure 1B). Our results also suggest that exposure of cancer cells to 2.5 µM of 5-FU for more than 24 h may result in even higher cytotoxicity (data not shown). The results of the bimonthly 48-h infusional protocol used by de Gramont et al seem to support this suggestion (Beerblock et al, 1997; de Gramont et al, 1997; 1998). These findings may form the bases for further exploration of the optimal dose/schedule for infusional 5-FU treatment.

We conclude that 24-h exposure of gastric cancer cells to low-concentration of 5-FU, as compared with 30-min exposure to high-concentration of 5-FU, resulted in better suppression of free TS, higher degree of S-phase blockade, and enhanced cytotoxicity. The findings of these in vitro studies may help explain the improved clinical efficacy of HDFL regimens.

### ACKNOWLEDGEMENTS

This study was supported by core grants (DOH87-HR-525; DOH88-HR-829) of the Cancer Research Center of National Taiwan University College of Medicine from the Department of Health, Executive Yuan, Taiwan.

### REFERENCES

Árdalan B, Chua L, Tian E, Reddy R, Sridhar K, Benedetto P, Richman S, Lepaspi A, Waldman S, Morrell L, Feun L, Savaraj N and Livingstone A (1991) A phase II study of weekly 24-hour infusion with high-dose fluorouracil with leucovorin in colorectal carcinoma. *J Clin Oncol* **9**: 625–630

Banerjee D, Schnieders B, Fu JZ, Adhikari D, Zhao SC and Bertino JR (1998) Role of E2F-1 in chemosensitivity. *Cancer Res* **58**: 4292–4296

Beerblock K, Rinaldi Y, Andre T, Louvet C, Raymond E, Tournigand C, Carola E, Favre R, de Gramont A and Krulik M (1997) Bimonthly high dose leucovorin and 5-fluorouracil 48-hour continuous infusion in patients with advanced colorectal carcinoma. Groupe d'Etude et de Recherche sur les Cancers de l'Ovaire et Digestifs (GERCOD). *Cancer* **79**: 1100–1105

Cheng AL, Yeh KH, Lin JT, Hsu C and Liu MY (1998) Cisplatin, etoposide, and weekly high-dose 5-fluorouracil and leucovorin infusion (PE-HDFL) – a very effective regimen with good patient compliance for advanced gastric cancer. *Anticancer Res* **18**: 1267–1272

Chomczynski P and Sacchi N (1987) Single-step method of RNA isolation by acid guanidinium thiocyanate-phenol-chloroform extraction. *Anal Biochem* **162**: 156–159

Chu E and Allegra CJ (1996) The role of thymidylate synthase in cellular regulation. *Adv Enzyme Regul* **36**: 143–163

Chu E, Drake JC, Koeller DM, Zinn S, Jamis-Dow CA, Yeh GC and Allegra CJ (1991*a*) Induction of thymidylate synthase associated with multidrug resistance in human breast and colon cancer cell lines. *Mol Pharmacol* **39**: 136–143

Chu E, Koeller DM, Casey JL, Drake JC, Chabner BA, Elwood PC, Zinn S and Allegra CJ (1991*b*) Autoregulation of human thymidylate synthase messenger RNA translation by thymidylate synthase. *Proc Natl Acad Sci USA* **88**: 8977–8981

de Gramont A, Bosset JF, Milan C, Rougier P, Bouche O, Etienne PL, Morvan F, Louvet C, Guillot T, Francois E and Bedenne L (1997) Randomized trial comparing monthly low-dose leucovorin and fluorouracil bolus with bimonthly high-dose leucovorin and fluorouracil bolus plus continuous infusion for advanced colorectal cancer: a French intergroup study. *J Clin Oncol* **15**: 808–815

de Gramont A, Louvet C, Andre T, Tournigand C and Krulik M (1998) A review of GERCOD trials of bimonthly leucovorin plus 5-fluorouracil 48-h continuous infusion in advanced colorectal cancer: evolution of a regimen. Groupe d'Etude et de Recherche sur les Cancers de l'Ovaire et Digestifs (GERCOD). *Eur J Cancer* **34**: 619–626

Dolnick BJ, Black AR, Winkler PM, Schindler K and Hsueh CT (1996) rTS gene expression is associated with altered cell sensitivity to thymidylate synthase inhibitors. *Advan Enzyme Regul* **36**: 165–180

Fraile RJ, Baker LH, Buroker TR, Horwitz J and Vaitkevicius VK (1980) Pharmacokinetics of 5-fluorouracil administered orally, by rapid intravenous and by slow infusion. *Cancer Res* **40**: 2223–2228

Grem JL (1996) 5-Fluoropyrimidines. In *Cancer Chemotherapy and Biotherapy: Principles and Practice*, Chabner BA and Longo DL (eds), pp 149–212. Philadelphia: Lippincott-Raven Publishers

Grem JL, McAtee N, Murphy RF, Balis FM, Steinberg SM, Hamilton JM, Sorensen JM, Sartor O, Kramer BS, Goldstein LJ, Gay LM, Caubo KM, Goldspiel B and Allegra CJ (1991) A pilot study of interferon alfa-2a in combination with fluorouracil plus high-dose leucovorin in metastatic gastrointestinal carcinoma. *J Clin Oncol* **9**: 1811–1820

Hsu CH, Yeh KH, Chen LT, Liu JM, Jan CM, Lin JT, Chen YC and Cheng AL (1997) Weekly 24-hour infusion of high-dose 5-fluorouracil and leucovorin in the treatment of advanced gastric cancers: an effective and low-toxic regimen for patients with poor general condition. *Oncology* **54**: 275–280

*© 2000 Cancer Research Campaign*

Johnston PG, Liang CM, Henry S, Chabner BA and Allegra CJ (1991) Production and characterization of monoclonal antibodies that localize human thymidylate synthase in the cytoplasm of human cells and tissue. *Cancer Res* **51**: 6668–6676

Johnston PG, Drake JC, Trepel J and Allegra CJ (1992) Immunological quantitation of thymidylate synthase using monoclonal antibody TS106 in 5-fluorouracil-sensitive and -resistant human cancer cell lines. *Cancer Res* **52**: 4306–4312

Johnston PG, Fisher ER, Rockette HE, Fisher B, Wolmark N, Drake JC, Chabner BA and Allegra CJ (1994) The role of thymidylate synthase expression in prognosis and outcome of adjuvant chemotherapy in patients with rectal cancer. *J Clin Oncol* **12**: 2640–2647

Johnston PG, Lenz HJ, Leichman CG, Danenberg KD, Allegra CJ, Danenberg PV and Leichman L (1995) Thymidylate synthase gene and protein expression correlate and are associated with response to 5-fluorouracil in human colorectal and gastric tumors. *Cancer Res* **55**: 1407–1412

Kohne CH, Schoffski P, Wilke H, Kaufer C, Andreesen R, Ohl U, Klaasen U, Westerhausen M, Hiddemann W, Schott G, Harstick A, Bade J, Horster A, Schubert U, Hecker H, Dorken B and Schmoll HJ (1998) Effective biomodulation by leucovorin of high-dose infusion fluorouracil given as a weekly 24-hour infusion: results of a randomized trial in patients with advanced colorectal cancer. *J Clin Oncol* **16**: 418–426

Laemmli UK (1970) Cleavage of structural proteins during the assembly of the head of bacteriophage T4. *Nature* **227**: 680–685

Lenz HJ, Leichman CG, Danenberg KD, Danenberg PV, Groshen S, Cohen H, Laine L, Crookes P, Silberman H, Baranda J and Garcia Y (1996) Thymidylate synthase mRNA level in adenocarcinoma of the stomach: a predictor for primary tumor response and overall survival. *J Clin Oncol* **14**: 176–182

Li W, Fan J, Hochhauser D, Banerjee D, Zielinski Z, Almasan A, Yin Y, Kelly R, Wahl GM and Bertino JR (1995) Lack of functional retinoblastinoma protein mediates increased resistance to antimetabolites in human sarcoma cell lines. *Proc Natl Acad Sci USA* **92**: 10436–10440

Meta-analysis Group In Cancer (1998) Efficacy of intravenous continuous infusion of fluorouracil compared with bolus administration in advanced colorectal cancer. *J Clin Oncol* **16**: 301–308

Navelgund LG, Rossana C, Muench AJ and Johnson LF (1980) Cell cycle regulation of thymidylate synthetase gene expression in cultured mouse fibroblasts. *J Biol Chem* **255**: 7386–7390

Peters GJ, van der Wilt CL, van Groeningen CJ, Smid K, Meijer S and Pinedo HM (1994) Thymidylate synthase inhibition after administration of fluorouracil with or without leucovorin in colon cancer patients: implications for treatment with fluorouracil. *J Clin Oncol* **12**: 2035–2042

Tsujinaka T, Kido Y, Shiozaki H, Iijima S, Homma T and Sakaue M (1992) Schedule-dependent inhibition of thymidylate synthase by 5-fluorouracil in gastric cancer. *Cancer* **70**: 2761–2765

Vanhoefer U, Wilke H, Weh HJ, Clemens M, Harstrick A, Stahl M, Hossfeld DK and Seeber S (1994) Weekly high-dose 5-fluorouracil and folinic acid as salvage treatment in advanced gastric cancer. *Ann Oncol* **5**: 850–851

Weh HJ, Wilke HJ, Dierlamm J, Klaassen U, Siegmund R, Illiger HJ, Schalhorn A, Kreuser ED, Hilgenfeld U, Steinke B, Weber W, Burkhard O, Zoller A, Pfitzner J, Subert R, Kriebel R and Hossfeld DK (1994) Weekly therapy with folinic acid (FA) and high-dose 5-fluorouracil (5-FU) 24-hour infusion in pretreated patients with metastatic colorectal carcinoma. *Ann Oncol* **5**: 233–237

Wilke H, Klaassen U, Achterrath W, Losch M, Vanhoefer U, Hayungs J, Harstrick A, Stahl M, Eberhardt W, Becher R and Seeber S (1996) Phase I/II study with a weekly 24-hour infusion of 5-fluorouracil plus high-dose folinic acid (HD-FU/FA) in intensively pretreated patients with metastatic breast cancer. *Ann Oncol* **7**: 55–58

Yeh KH and Cheng AL (1994) An alternative method to overcome central venous portable external infusion pump blockage in patients receiving weekly 24-hour high-dose fluorouracil and leucovorin. *J Clin Oncol* **12**: 875–876 (letter)

Yeh KH and Cheng AL (1998) Gastric cancer associated with acute disseminated intravascular coagulation – successful initial treatment with weekly 24-hour infusion of high-dose 5-fluorouracil and leucovorin. *Br J Haematol* **100**: 769–772

Yeh KH, Cheng AL, Lin MT, Hong RL, Hsu CH, Lin JF, Chang KJ, Lee PH and Chen YC (1997) A phase II study of weekly 24-hour infusion of high-dose 5-fluorouracil and leucovorin (HDFL) in the treatment of recurrent or metastatic colorectal cancers. *Anticancer Res* **17**: 3867–3872

Yeh KH, Shun CT, Chen CL, Lin JT, Lee WJ, Lee PH, Chen YC and Cheng AL (1998*a*) High expression of thymidylate synthase is associated with drug resistance of gastric cancers to high-dose 5-fluorouracil-based systemic chemotherapy. *Cancer* **82**: 1626–1631

Yeh KH, Chen YC, Yeh SH, Chen JP, Lin JT and Cheng AL (1998*b*) Detection of circulating cancer cells by nested reverse transcription-polymerase chain reaction of cytokeratin-19 (K19) – possible clinical significance in advanced gastric cancer. *Anticancer Res* **18**: 1283–1286

*© 2000 Cancer Research Campaign* 
